# Carbapenem-resistant hypervirulent *Klebsiella pneumoniae*: where is it headed in the tug-of-war between virulence and resistance?

**DOI:** 10.1016/j.ebiom.2025.105649

**Published:** 2025-03-10

**Authors:** Meng Wang, Hui Wang

**Affiliations:** a Department of Clinical Laboratory, Peking University People's Hospital, Beijing, China; b Institute of Medical Technology, Peking University Health Science Center, Beijing, China

The emergence of carbapenem-resistant hypervirulent *Klebsiella pneumoniae* presents serious challenge to global public health.^1^ Traditionally, hypervirulent *K. pneumoniae* (hvKP) and carbapenem-resistant *K. pneumoniae* (CRKP) occupied distinct ecological niches: hvKP strains, associated with community-acquired infections such as liver abscesses and endophthalmitis, retained antibiotic susceptibility despite their high virulence, while CRKP strains, primarily responsible for hospital-acquired pneumonia and sepsis, exhibited multidrug resistance but were less virulent.^2^ However, this dichotomy has been disrupted through two convergent evolutionary pathways: 1) CRKP acquiring virulence plasmids (e.g., the ST11-KL64 clone, now epidemic in China), resulting in hv-CRKP strains with enhanced virulence, though still less virulent than classical hvKP^3^; 2) Classical hvKP lineages (e.g., ST23-K1, ST86-K2) acquiring carbapenemase plasmids, forming CR-hvKP with rising prevalence, while the thick capsule hampers plasmid uptake, and resistance acquisition is often comes with CPS gene mutations that reduce virulence.^4^ Crucially, these processes require balancing plasmid stability, capsule biosynthesis, CRISPR-Cas system activity, and metabolic burden, making it difficult for high virulence and strong resistance phenotypes to coexist.^5^

A recent study by Zhang et al. in *eBioMedicine* highlights the pivotal role of the IncFII_K34_ KPC-2 plasmid in reshaping this evolutionary landscape.^6^ Found in 25% of KPC-producing CR-hvKP strains across 24 countries, this plasmid overcomes barriers through a three-pronged adaptive strategy of “efficient conjugation, enhanced resistance, and sustained virulence”. First, it upregulates conjugation-related genes such as the tra operon (e.g., *traJ* and *traM*), boosting transfer efficiency by several orders of magnitude compared to traditional IncFII_K2_ plasmids. Second, IncFII_K34_ confers a growth advantage to its host under meropenem pressure by increasing the copy number and expression of the *bla*_KPC_ gene, creating a self-reinforcing “resistance-transmission” cycle. Alarmingly, the plasmid enables a “stealth virulence” phenotype: CR-hvKP strains lose hypermucoviscosity while retain lethal hypervirulence in both murine and human neutrophil models, indicating that hypervirulence and hypermucoviscosity may be independently regulated rather than strictly linked.

The evolutionary trajectory of CR-hvKP forces a reckoning with the complex interplay between resistance and virulence in the post-antibiotic era. Plasmids such as IncFII_K34_ and IncFIB(Mar), now recognised as pivotal vectors for disseminating hybrid resistance-virulence gene networks,^7^ challenging conventional surveillance paradigms centred on clonal typing (e.g., MLST, cgMLST). A shift toward plasmid-centric surveillance is essential to track mobile genetic elements driving the synergistic evolution of resistance and virulence. In particular, highly transmissible vectors must be closely monitored to kerb their further spread, integrating plasmid dynamics into genomic surveillance and accelerating the development of rapid diagnostics for resistance-virulence hybrids. Additionally, hvKP diagnostic protocols need also evolve, the dissociation of hypervirulence from hypermucoviscosity challenges the effectiveness of traditional phenotypic assays used for hvKP detection. Instead, molecular diagnostics targeting key virulence loci (*iucA*, *iroB*, *peg-344*, *rmpA*, and *rmpA2*), carbapenemase genes, and K locus typing must be integrated into routine surveillance programs to enhance strain identification and epidemiological tracking.

From a therapeutic standpoint, addressing CR-hvKP requires a multifaceted approach. Kerbing carbapenem overuse remains a cornerstone strategy to minimise selection pressure and slow the emergence of resistance. However, given the limited efficacy of current antibiotic regimens against CR-hvKP, alternative therapeutic interventions are urgently needed. Phage therapy, which targets bacterial pathogens with bacteriophages, presents a promising avenue for precision treatment.^8^ Similarly, anti-virulence strategies aimed at disarming key virulence factors without exerting selective pressure for resistance offer a novel approach.^9^ Furthermore, synthetic biology tools for plasmid clearance and CRISPR-Cas-based genome editing hold potential for eradicating resistance elements at the genetic level.^10^

In conclusion, the evolution of CR-hvKP exemplifies the dynamic interplay between bacterial virulence and antimicrobial resistance. Rather than existing as distinct traits, virulence and resistance are shaped by plasmids as modular platforms that enhance pathogen adaptability across environments. The rising threat of CR-hvKP, which bridges the gap between community and hospital pathogens, necessitates a paradigm shift in surveillance and treatment strategies. Integrating molecular diagnostics, plasmid-based surveillance, and innovative therapeutics will be critical to anticipating and combating this formidable public health threat.

## Contributors

Literature search, writing, and editing: Meng Wang and Hui Wang. Both authors read and approved the final manuscript.

## Declaration of interests

The authors declare no conflict of interest.
